# Lipid accumulation drives cellular senescence in dopaminergic neurons

**DOI:** 10.18632/aging.206030

**Published:** 2024-07-19

**Authors:** Taylor Russo, Markus Riessland

**Affiliations:** 1 Department of Neurobiology and Behavior, Stony Brook University, Stony Brook, NY 11794, USA; 2 Center for Nervous System Disorders, Stony Brook University, Stony Brook, NY 11794, USA

**Keywords:** lipids, cellular senescence, Parkinson’s disease, glucosylceramides, lysosomes, neuroinflammation

## Abstract

Parkinson’s disease (PD) is an age-related movement disorder caused by the loss of dopaminergic (DA) neurons of the substantia nigra pars compacta (SNpc) of the midbrain, however, the underlying cause(s) of this DA neuron loss in PD is unknown and there are currently no effective treatment options to prevent or slow neuronal loss or the progression of related symptoms. It has been shown that both environmental factors as well as genetic predispositions underpin PD development and recent research has revealed that lysosomal dysfunction and lipid accumulation are contributors to disease progression, where an age-related aggregation of alpha-synuclein as well as lipids have been found in PD patients. Interestingly, the most common genetic risk factor for PD is Glucosylceramidase Beta 1 (GBA), which encodes a lysosomal glucocerebrosidase (GCase) that cleaves the beta-glucosidic linkage of lipids known as glucocerebrosides (GluCer). We have recently discovered that artificial induction of GluCer accumulation leads to cellular senescence of DA neurons, suggesting that lipid aggregation plays a crucial role in the pathology of PD by driving senescence in these vulnerable DA neurons. Here, we discuss the relevance of the age-related aggregation of lipids as well as the direct functional link between general lipid aggregation, cellular senescence, and inflammaging of DA neurons. We propose that the expression of a cellular senescence phenotype in the most vulnerable neurons in PD can be triggered by lysosomal impairment and lipid aggregation. Importantly, we highlight additional data that perilipin (PLIN2) is significantly upregulated in senescent DA neurons, suggesting an overall enrichment of lipid droplets (LDs) in these cells. These findings align with our previous results in dopaminergic neurons in highlighting a central role for lipid accumulation in the senescence of DA neurons. Importantly, general lipid droplet aggregation and global lysosomal impairment have been implicated in many neurodegenerative diseases including PD. Taken together, our data suggest a connection between age-related lysosomal impairment, lipid accumulation, and cellular senescence in DA neurons that in turn drives inflammaging in the midbrain and ultimately leads to neurodegeneration and PD.

## INTRODUCTION

**Figure 1:**
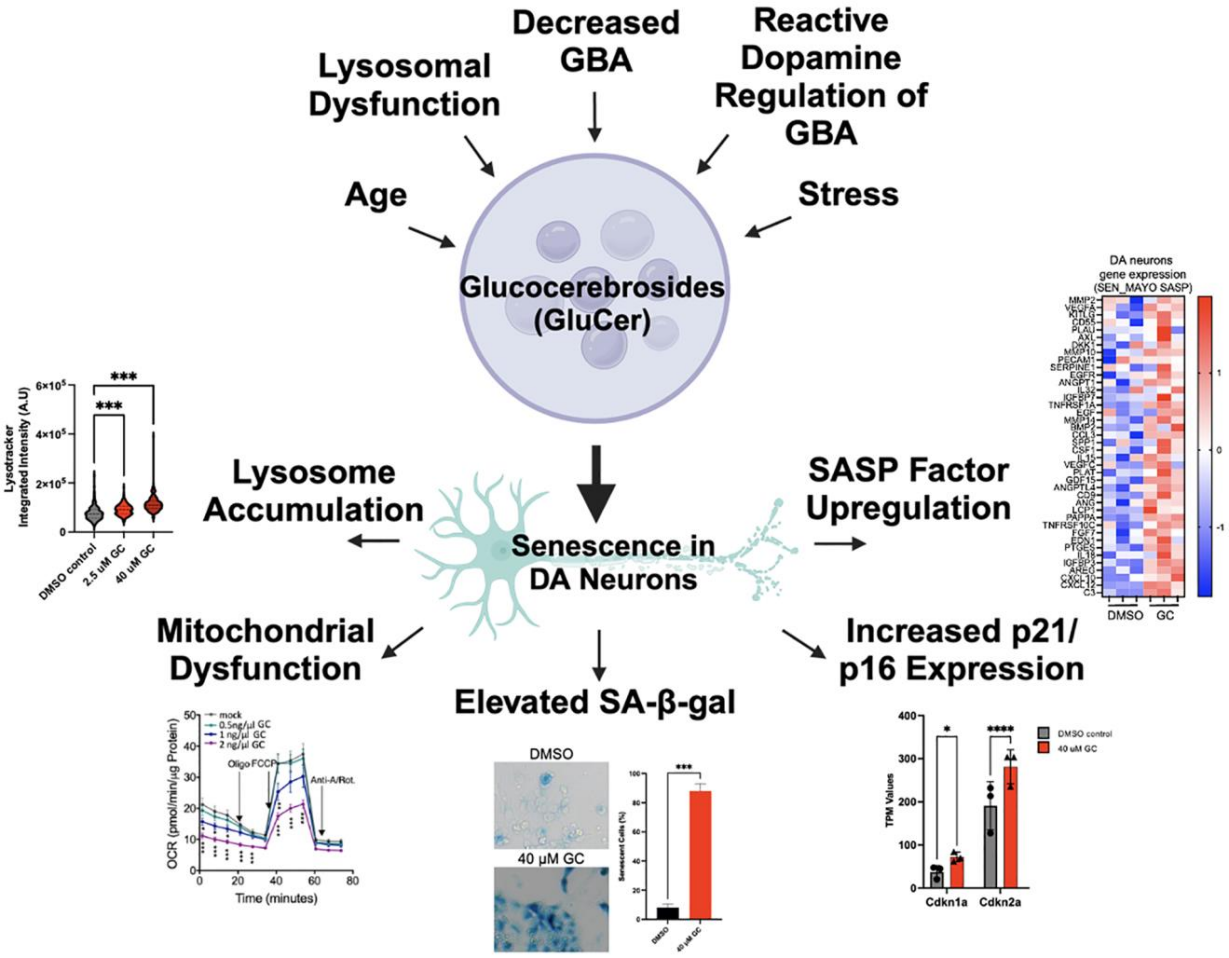
**Overview of drivers of GluCer accumulation, which leads to cellular senescence in dopaminergic neurons.** Aging, stress, lysosomal dysfunction, decreased levels of GBA, and the regulation of GBA by reactive dopamine have all been shown to lead to the accumulation of glucocerebrosides (GluCer). We have recently shown that the accumulation of GluCer directly drives senescence of dopaminergic (DA) neurons, indicated by lysosomal accumulation, mitochondrial dysfunction, elevated senescence-associated ß-galactosidase, increased p21/p16 expression, and SASP factor upregulation (representative diagrams and images are modified from our previous study [10]).

The age-related neurodegenerative movement disorder Parkinson’s disease (PD) is characterized by the specific and progressive loss of dopaminergic (DA) neurons in the substantia nigra pars compacta (SNc) [1]. The selective loss of these vulnerable DA neurons results in the hallmark motor symptoms of PD, including tremors, rigidity, and bradykinesia. Importantly, non-motor symptoms, such as cognitive impairment, depression, constipation, and sleep problems, among others, are also associated with the disease. In addition to these symptoms, a key pathological hallmark of PD is the intracellular aggregation of alpha-synuclein (a-SYN), leading to the formation of Lewy bodies. a-SYN has been shown to alter lipid metabolism in PD, and interestingly the accumulation of lipids has been shown to result from a variety of PD-inducing stressors [2]. While aging is the most significant risk factor for PD, genetic predispositions can also contribute to the etiology of the disease. The most common genetic risk factor for PD is GBA, which encodes the lysosomal enzyme β-glucocerebrosidase (GCase) that is crucial for the breakdown of specific lipids known as glucosylceramides (GluCer). While homozygous mutations in GBA cause the most common lysosomal storage disorder Gaucher’s disease, heterozygous variations in this gene are associated with PD [3, 4]. In line with this, it has been shown that levels and activity of GCase are associated with aging and sporadic forms of PD, emphasizing the role of GCase in neuronal vulnerability [5, 6]. While reduced activity and/or levels of GCase causes aggregation of GluCer, it has been reported that increased levels of GluCer in the cerebrospinal fluid of PD patients correlate with rapid cognitive decline, which further implicates lipid dysregulation in the pathology of PD [5]. There is evidence that GluCer accumulation can occur as the result of not only aging or decreased levels/activity of GCase, but also in models of lysosomal dysfunction [7] and downstream of the regulation of GBA by reactive dopamine [8, 9] (Figure 1).

Importantly, our recent research unraveled a molecular basis for DA neuron impairment in the context of PD, as we delineated a novel pathway connecting age-related GBA decline to cellular senescence [10]. Specifically, we found that the risk factor for PD and genetic master regulator SATB1 controls the expression of micro-RNA miR-22-3p, which in turn regulates GBA expression. The reduction of SATB1 results in the de-repression (upregulation) of miR-22-3p, leading to decreased GBA levels, accumulation of GluCer, and the promotion of cellular senescence in DA neurons. Importantly, it has been shown that the expression of both GBA and SATB1 declines with age, while miR-22-3p expression increases [11]. Additionally, recent studies link reduced GCase activity to senescence in lysosomal disorders [12]. These findings, together with evidence of age-related GluCer accumulation and a general increase of inflammaging and the senescence burden with age, suggest that lipid-accumulation might be directly linked to cellular senescence (Figure 1). To evaluate the hypothesis that GluCer may directly drive cellular senescence in DA neurons, we treated human wild-type DA neurons as well as DA neuroblastoma cell lines with GluCer and found that this was sufficient to induce a senescence phenotype. In addition to inducing senescence, GluCer also triggered a-SYN aggregation, further linking it to the PD pathology [10]. In summary, we were able to establish that the accumulation of GluCer was sufficient to induce a variety of key senescence phenotypes in DA neurons, including lysosome accumulation, mitochondrial dysfunction, elevated senescence-associated ß-galactosidase (SA-ß-gal) activity, increased p21/p16 levels, and the upregulation of senescence-associated secretory phenotype (SASP) factor expression (Figure 1).

In line with our findings related to lipid-induced senescence, both cell line and animal models have shown that accumulation of lipid droplets (LD) leads to increased alpha-synuclein accumulation both by direct interaction and synergistic aggregation. Additionally, cellular senescence has been shown to be associated with the accumulation of LD [13]. Together these findings, along with our results showing GluCer-induced DA neuron senescence, suggest that general lipid aggregation plays a crucial role in the pathology of PD. While the direct link between lipids and loss of DA neurons remains elusive, recent data suggests that lipid accumulation can drive senescence resulting in progressive loss of functional neurons. Interestingly, LD formation is directly linked to the function of perilipin 2 (PLIN2), a protein that is expressed in neurons and upregulated in the aging brain [14]. In fact, overexpression of PLIN2 is sufficient to induce formation of LDs. However, it remains unclear whether both PLIN2 and LDs correlate with cellular senescence. Some studies suggest that LD formation could be seen as a hallmark of senescence because it is mainly triggered by lysosomal impairment, which is a well-established feature of senescent cells. Interestingly, however, LDs have been shown to trigger a-SYN aggregation which can later lead to the formation of Lewy bodies. In line with this, the artificial induction of the aggregation of a-SYN has been shown to be sufficient to drive neuronal senescence [15]. Thus, it is plausible that LDs could indeed contribute to driving senescence, possibly in a cell type-specific manner. Many recent studies suggest that the process of cellular senescence itself is a highly cell type-specific process, whereby the phenotypes/features of senescence may differ as well as the general capacity and/or vulnerability of a given cell type to enter a state of senescence. Especially for post-mitotic neurons, it appears that some neuron types are prone to enter a state of cellular senescence, whereas others are less prone to a given senescence-driving insult. For example, the reduction of the function of master regulator SATB1 in DA neurons triggered an upregulation of p21 and drove cellular senescence. In comparison however, the same knockout in cortical stem cell-derived neurons did not have an impact on p21 levels and therefore did not trigger cellular senescence, demonstrating the cell type-dependent nature of senescence induction [16]. Recent research in Alzheimer’s disease describes that cellular senescence is specifically triggered in neurons of the hippocampus and entorhinal cortex [17, 18]. Interestingly, DA neurons, similarly to entorhinal cortex neurons, appear to depend heavily on the function of lysosomes. In fact, lysosomal dysfunction or mutations in a variety of lysosomal genes significantly elevate the likelihood of PD, possibly by driving a-SYN aggregation.

**Figure 2:**
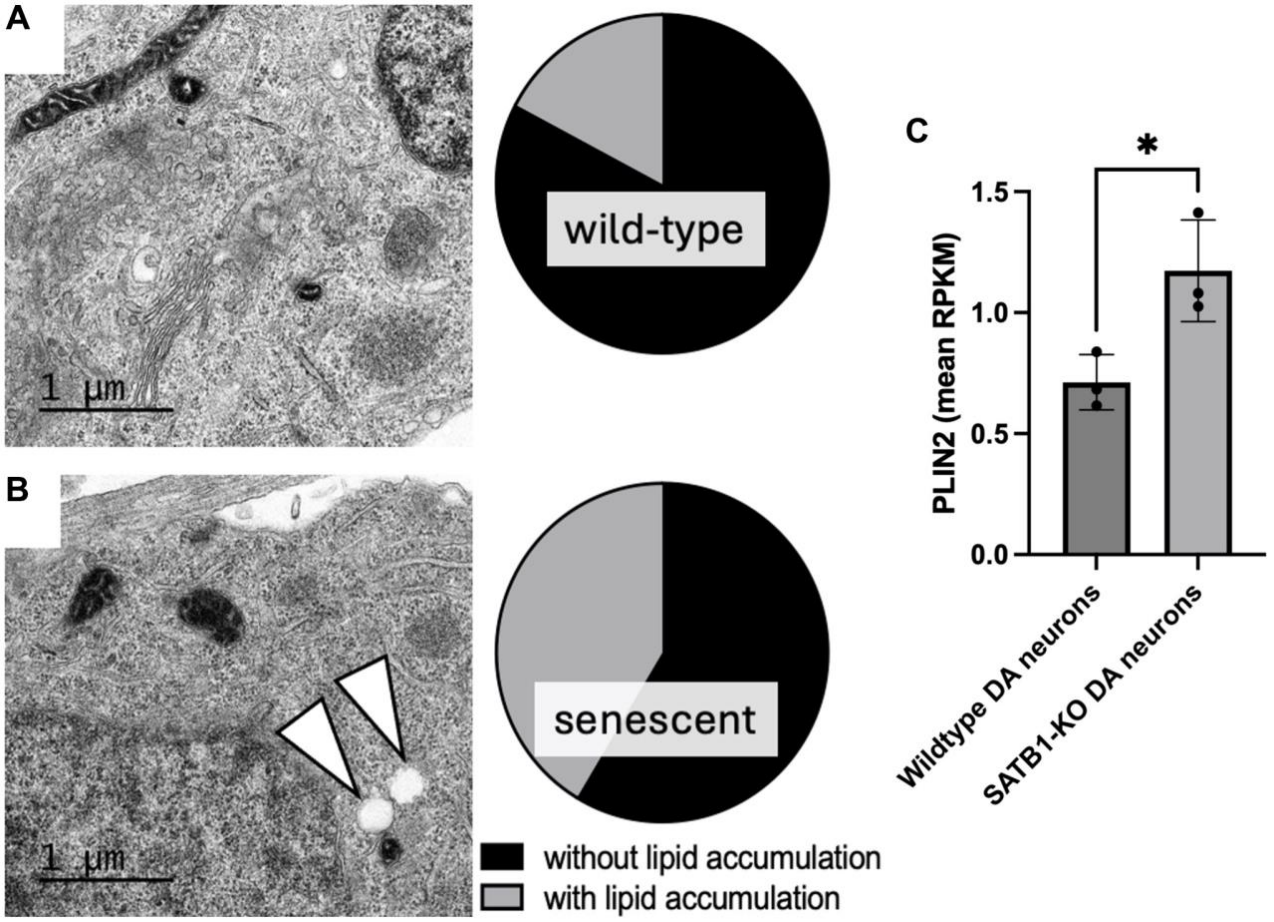
**Quantification of lipid accumulation and PLIN2 expression in human stem cell derived DA neurons.** Representative TEM images of a mature wild-type (**A**) and SATB1-KO (senescent) DA neurons (**B**). Pie charts show the quantification of cells containing LD-like structures. White arrowheads indicate LD-like structures. *N* = 3, *n* = 29 per genotype. Scale bar = 1 um. (**C**) PLIN2 expression in wildtype and SATB1-KO (senescent) DA neurons. Student’s *t*-test was performed. ^*^p < 0.05.

Given the connection between LDs and a-SYN, it is plausible that there is an overlap between PD driving pathways and age-related lysosomal dysfunction as well as neuronal senescence. Global lysosomal impairment has been implicated in many neurodegenerative diseases including PD [19]. Generally, DA neurons are selectively vulnerable to lysosomal dysfunction. Thus, LDs may be prone to form more readily due to lysosomal impairment and consequently may drive the cellular senescence of DA neurons leading to midbrain inflammaging and eventually PD. Interestingly, we observe LD-like structures in transmission electron microscopic (TEM) images of SATB1 knockout (senescent) human stem cell-derived DA neurons (Figure 2). In line with this, we also observe a significant upregulation of PLIN2 in senescent (SATB1 knockout) DA neurons, suggesting an activation of the pathway driving LD formation. In summary, our data shows that GluCer directly elicits cellular senescence in DA neurons and suggests that PLIN2 upregulation in senescent cells may worsen the lipid accumulation, possibly driving a-SYN accumulation, and reinforce the senescence phenotype as well as inflammaging and thereby immune reaction-dependent DA neuron loss in PD. Since the role of the connection between LDs and cellular senescence in neurons is understudied, it would be intriguing to further unravel the relationship between the two, particularly in the context of aging, PD, and neurodegeneration. Further understanding of this pathway, along with the role of lipids in aging and inflammaging in the context of cellular senescence, has the potential to open new avenues for potential intervention for a variety of devastating neurodegenerative disorders, such as PD and Alzheimer’s disease.
